# Academy of Medicine — Erie County Medical Society

**Published:** 1911-06

**Authors:** 


### Academy of Medicine – Erie County Medical Society

A PUBLIC meeting under the auspices of the Erie County Medical Society and the Buffalo Academy of Medicine was held at the Central Y. M. C. A. auditorium on Monday evening, May 15, at which Dr. J. N. McCormack, chairman of the organisation committee of the American Medical Association, spoke on "Things about doctors which doctors and other people ought to know." Mayor Fuhrmann, of Buffalo, presided and addresses were delivered by the president of the Chamber of Commerce; Hon. H. P. Bissell, of the New York State Lunacy Commission and Rev. F. T. Parr, rector of St. Mary's Catholic Church.

Dr. McCormack is a forceful talker and his address generally dealt with conservation of health. He urged the formation of county societies to hold joint meetings of physicians with women's clubs, teachers' organisations, editors, lawyers, clergymen, business men's associations and labor unions for the discussion of matters of mutual interest. He also urged a movement looking to the establishment of a national board of health which would have general supervision over the whole country. The conservation of public health, he declared was one of the greatest assets of the nation. "The drain upon this asset," he said, "from diseases known to be preventable, and as a result of vicious and immoral living, is estimated to be more than one third of the entire sick and death rate every year.

"You've got to be a horse or a hog or a cow to get any financial assistance from the government," he said and then quoted that in ten years our national government has expended $40,000,000 and now proposes to spend $250,000,000 to prevent tick fever in cattle, cholera in hogs and chickens, pests to crops and trees and to protect other interests having money value, "while in all its history it has never spent a dollar or lifted a hand to protect the people from tuberculosis, typhoid fever, diphtheria, measles, scarlet fever and other preventable diseases of humans."

"As an argument for peace, it is shown that 210,000 men were killed in both armies during the five years of the civil war. As an argument for healthier living, I want to show you that 750,000 persons have died from tuberculosis in the last five years and that about 1,000,000 are constantly ill with it; that there were 250,000 deaths and 2,500,000 persons ill in the same period from preventable diseases.

"Tuberculosis is not inherited. It is caught, and if all the expectorated matter from every case now in this state could be destroyed, as your health officials and physicians are earnestly trying to do, until all now ill either recover or die, and other necessary precautions be taken, you would soon have none but imported cases.

"Typhoid fever is a filth disease. No one can have it except by getting into the mouth something from the bowels or kidneys of someone who has the disease, usually carried there by water, milk or flies. Cities must prevent pollution of their water supply; they must dispose of their sewage otherwise than by emptying it into streams. Health departments must exercise closer supervision of dairies. Flies must be and can be got rid of, and the time will come when it will be a reproach to a woman to have them in her home.

"Physicians must keep up with their science, which is advancing in leaps and bounds. Post-graduate courses for them are being established; they should keep studying, attend medical conventions. They should keep in touch with the laity and set an example, in both home and professional life, of clean, moral, healthy living.

"The physicians and druggists should keep the people posted on deleterious patent medicines. Many women scorn to drink a glass of beer, containing five per cent. of alcohol, or of champagne, containing eight per cent. Yet they will drink cheap whiskies, sold at a high price, as medicines, and various patent concoctions that are cocktails, some of them flavored with drugs and others with burnt sugar.

"A health department at Washington, with laboratories and research workers in proportion to our power and wealth and the vast interests involved, to investigate the cause of the common diseases not yet understood, to gather, tabulate and make public the facts in regard to every case of sickness and death which occurs in every county and state, with prevention as the only object, is as much of a necessity, if our people are to have the benefactions of modern science utilised daily for the protection of their homes and families, as is the Supreme Court of the United States."

Dr. McCormack advocated vocational training schools, domestic science teaching in the schools, sex education for the young and the holding of neighborhood meetings for the discussion of subjects to improve health and morals.

---

At the regular meeting of section on surgery held May 2, the following officers were elected: Chairman, Dr. James H. Lewis; Secretary, Dr. Herbert A. Smith.

No further business of the section was transacted. A special meeting of the Academy followed to take action on the death of the president, Dr. Carlton C. Frederick.

At the regular meeting of the section on medicine, held May 9, the following officers were elected: Chairman, Dr. Henry C. Buswell; Secretary, Dr. Carl S. Tompkins.

Dr. Buswell read a paper on Oidiomycosis: A report of an unusual case.

At the regular meeting of the section on pathology, held May 16, the following officers were elected: Chairman, Dr. Henry Adsit; Secretary, Dr. Guy L. McCutcheon.

Dr. Joseph Lewis read a paper for Dr. DeWitt H. Sherman on Lumbar Puncture and Dr. A. A. Thibaudeau read a paper on the Wasserman reaction.

At the regular meeting of the section on obstetrics and gynecology, held May 23, the following officers were elected: Chairman, Dr. Joseph S. Lewis; Secretary, Dr. Alfred Noehren.

